# A possible association between dysphonia and sleep duration: A cross-sectional study based on the Korean National Health and nutrition examination surveys from 2010 to 2012

**DOI:** 10.1371/journal.pone.0182286

**Published:** 2017-08-04

**Authors:** Jung-Hae Cho, Christian Guilminault, Young-Hoon Joo, Sang-Kyun Jin, Kyung-Do Han, Chan-Soon Park

**Affiliations:** 1 Department of Otolaryngology-Head and Neck Surgery, College of Medicine, The Catholic university of Korea, Seoul, Republic of Korea; 2 Center for Sleep Medicine, Department of Psychiatry and behavioral science, Stanford University, Redwood City, CA, United States of America; 3 Department of Biostatistics, College of Medicine, The Catholic University of Korea, Seoul, Republic of Korea; Charité - Universitätsmedizin Berlin, GERMANY

## Abstract

**Background:**

Sleep is important in terms of good general health and appropriate sleep duration has been linked to quality-of-life. Dysphonia may impair communication and social relationships, and is thus also closely related to quality-of-life. No large-scale, cross-sectional epidemiological study of a sample representative of the population of an entire country has yet assessed the possible existence of a relationship between sleep duration and dysphonia.

**Methods:**

We investigated a possible association between subjective voice problems and self-reported sleep duration in South Korean subjects using 2010–2012 data from the Korean National Health and Nutrition Examination Survey (KNHANES). Cross-sectional data on 17,806 adults (7,578 males and 10,228 females) over the age of 19 years who completed the KNHANES were analyzed. All participants reported voice problems (if present) and their daily average sleep duration using a self-reporting questionnaire. Sleep duration was classified into five categories as follows: ≤5, 6, 7, 8, and ≥9 h/day.

**Results:**

The overall prevalence of dysphonia was 6.8%; 5.7% in males and 7.7% in females. The prevalence for dysphonia by sleep duration exhibited a U-shape, with the lowest point being at sleep duration of 7-8h. After adjustment for covariates (age, sex, smoking status, alcohol consumption, regular exercise, low income, high-level education), a sleep duration of ≤5 h (OR = 1.454; 95% CI, 1.153–1.832) and a sleep duration of ≥9 h (OR = 1.365; 95% CI, 1.017–1.832) were significantly associated with dysphonia, compared to a sleep duration of 7 h. In terms of gender, males who slept for ≥9 h were at a 2-fold (OR = 2.028; 95% CI, 1.22–3.35) higher odds for dysphonia (p<0.05) compared to those who slept for 7 h. A sleep duration ≤5 h was associated with a 1.6-fold (OR = 1.574; 95% CI, 1.203–2.247) higher odds of dysphonia ≥3 weeks in duration (long-term dysphonia).

**Conclusions:**

This is the first study to show that both short and long sleep duration were significantly associated with the development of dysphonia. The association between sleep duration and dysphonia was more marked in males than females. A sleep duration ≤5 h had a significant impact on the prevalence of long-term dysphonia.

## Introduction

Sleep duration is associated with quality-of-life and general health outcomes [1, 2]. Previous studies have shown that unusually short or long sleep durations are associated with mortality from cardiovascular disease, hypertension, obesity, and cancer [2–6]. As extreme sleep durations (abnormally long or short) affect health and morbidity, the ideal sleep duration is considered to be 6–8 h per day. Recent studies performed in Korea and Spain have suggested that a sleep duration of 7–8 h should be recommended to maintain a generally healthy lifestyle [7, 8]. In addition, Chinese studies have found relationships between sleep duration and quality-of-life in adolescents and adults, and between sleep deprivation and health-related quality-of-life in older adults [9, 10]. These studies showed that sleep was closely related to quality-of-life, as is voice.

A national health survey conducted in the USA found that about 7.6% of adults reported a voice problem annually [11]. The cumulative frequency of such problems over a life-span is thought to be much higher. Risk factors for dysphonia have been extensively investigated [12, 13]. Well-known common causes of voice problems are: voice overuse or misuse, lifestyle choice or occupation, environmental pollution, pharmacological agents or alcohol, laryngitis or laryngopharyngeal reflux, and a neural disorder of the larynx. These factors, alone or in combination, affect the voice. In addition, dysphonia may be either temporary or persistent, depending on the etiology. In particular, dysphonia that lasts for >3 weeks (long-term dysphonia) is often caused by laryngeal pathologies and must be thoroughly evaluated [14].

It is generally considered that the voice changes when sleep duration is inadequate; fatigue affects voice. Sleep deprivation may affect the ability to voice feeling and emotion, emphasizing the importance of sleep in terms of healthy adult emotional functioning [15]. Moreover, patients with dysphonia had a lower voice-specific quality-of-life and higher depression scores [13]. Therefore, maintenance of good voice quality is an important component of good general health.

Based on previous studies, we hypothesized that inappropriate sleep duration, either too short or too long, would be associated with poor voice quality. However, to the best of our knowledge, no population-based study has yet examined the relationship between dysphonia and sleep duration in adults. Therefore, our aim was to investigate the relationship between self-reported sleep duration and dysphonia in Korean subjects using the extensive data available on the national prevalences of various diseases.

## Materials and methods

### Ethic statement

Written informed consent was obtained from all participants prior to the survey, and approval was obtained from the Institutional Review Board of the Catholic University of Korea in Seoul, South Korea.

### Study population

Data were obtained from the Korean National Health and Nutrition Examination Survey(KNHANES); this is a cross-sectional survey designed to measure the health and nutritional status of the non-institutionalized Korean population from 2010 to 2012. KNHANES is a government-sponsored survey conducted by the Korean Center for Disease Control and Prevention, and commenced in 1998. Individuals were selected annually and complete questionnaires exploring health status, health behavior, nutrition, and attendance at health examinations. Participating households were selected with the aid of a stratified, multistage probability sampling design. The survey featured a health interview, nutritional assessment, and a health examination. Demographic data and information on health-related behaviors were collected by self-reported questionnaire and during personal interviews. Medical staff conducted physical examinations and blood and urine sampling using standard procedures. All participants provided written informed consent.

### Subjective voice problems and sleep duration

Participants aged ≥19 years were studied; they were asked about subjective vocal problems and attended otolaryngyological interviews. Self-reported vocal problems were classified as “present” or “absent” depending on the response to the question: “Do you currently experience voice pain and/or discomfort?” Participants who responded positively were then asked: “Have you had this problem for 3 weeks or longer?” Long-term dysphonia was defined as dysphonia persisting for ≥3 weeks. Question for self-reported voice problems was designed by the Epidemiologic Survey Committee of the Korean Otolaryngologic Society. In addition, the Korea centers for disease control and prevention verified the quality of the survey. Sleep duration was self-reported. All participants were asked: “How long do you usually sleep every night?” The responses were classified as ≤5, 6, 7, 8, and ≥9h.

### Demographic variables

Medical histories and lifestyle habits were self-reported. Smoking history was categorized as current smoker, ex-smoker, and nonsmoker. Based on the amount of alcohol consumed per day during the 1-month period prior to the interview, all subjects were classified into three groups: nondrinkers, mild-to-moderate drinkers (<15 g alcohol/day), and heavy drinkers (≥15 g/day). Regular exercise was defined as strenuous physical activity performed for a minimum of 20 min three times a week. Marital status was classified as with or without a spouse. Occupation was divided into working and not working. Residential area was defined as urban or rural; urban included both large and small cities. Educational level was classified as high if the respondent had completed university education. Household incomes were divided into four quartiles.

### Anthropometric and laboratory measurements

Weight and height were measured by well-trained medical professionals. Standing height was measured with each subject facing directly ahead, with shoes off, the feet together, the arms at the sides, and the heels, buttocks, and upper back in contact with the wall. Height was measured in cm to the nearest mm using a SECA 225 (Germany). Waist circumference (WC) was measured to the nearest mm at the level of the midpoint between the iliac crest and the costal margin, at the end of a normal expiration. Weight was measured in kg, to the nearest 10 g, using a GL-6000-20 scale (Cass Korea, Seoul, Korea). Body mass index(BMI) was calculated as weight (kg)/height (m^2^). Blood pressure (BP) was measured with each subject in a sitting position after a 5-min rest period. Systolic blood pressure (SBP) and diastolic blood pressure (DBP) were measured on the right arm using a mercury sphygmomanometer (Baumanometer, W.A. Baum Co., Copiague, NY, USA). Systemic hypertension was defined as a systolic blood pressure >140 mmHg and/or a diastolic blood pressure >90 mmHg, or current use of systemic antihypertensive drugs. Blood samples were obtained from the antecubital vein following a 10–12 h (overnight) fast. The serum levels of glycemia, total cholesterol (TC), triglycerides (TG), high-density lipoprotein (HDL) cholesterol, and low-density lipoprotein (LDL) cholesterol, were measured using enzymatic methods (Hitachi Automatic Analyzer 7600, Hitachi, Tokyo, Japan). Diabetes was defined as a fasting blood glucose level >126 mg/dL or current use of antidiabetic medication. Metabolic syndrome (MetS) was considered present if at least three of the following criteria were met: WC ≥90 cm in males or ≥80 cm in females (the modified Asian criteria); TG ≥150 mg/dL or prescription of TG-lowering medication; a reduced level of HDL cholesterol (<40 mg/dL in males or <50 mg/dL in females); an SBP ≥130 mmHg, a DBP ≥85 mmHg, or use of antihypertensive medication; and an FBS level ≥100 mg/dL or use of anti-diabetes medicationor previously diagnosed diabetes mellitus [16]. We also measured serum creatinine levels and calculated estimated glomerular filtration rates(eGFRs; mL/min/1.73m^2^) using the following equation from the Chronic Kidney Disease Epidemiology Collaboration (CKD-EPI): eGFR = 141 × min(serum creatinine/κ,1)^α^ × max(serum creatinine/κ,1)^−1.209^ × 0.993^age^ × 1.018 [if female] × 1.159 [if African-American], where κ was 0.7 for females and 0.9 for males; α was -0.411 for males and -0.329 for females; min indicates minimum serum creatinine/κ, or 1; and max indicates maximum serum creatinine/κ or 1 [17]. Chronic kidney disease was considered present when the eGFR was <60mL/min/1.73m^2^.

### Statistical analysis

All statistical analyses were performed using the survey module of Statistical Analysis Software (SAS) (v. 9.3; SAS Institute, Cary, NC, USA); this was appropriate, considering the complex sample design and sampling weights of KNHANES. Again, KNHANES seeks to derive nationally representative data. All continuous variables are given as means with standard errors (SEs), and all categorical variables as numbers with percentages. The Rao-Scott chi-squared test or Student’s *t*-test (run using the PROC SURVEYFREQ module of SAS) and logistic regression analysis (using the PROC SURVEYLOGISTIC module of SAS) were employed to explore associations between dysphonia and various risk factors using a complex sampling design. Upon multiple logistic regression analysis, we first adjusted for age and sex (model 1); next for these variables plus BMI (model 2); and finally for the variables of model 1 plus smoking status, alcohol intake, regular exercise, income, and educational level (model 3).The prevalences of dysphonia (with 95% confidence intervals [CIs]) were calculated. All *p*-values were two-tailed and a *p* value <0.05 was considered to reflect statistical significance.

## Results

### General characteristics of the study population

Subject demographics are summarized in Table 1. Of the 17,806 participants aged ≥19 years, 1,218(6.8%) complained of dysphonia. The mean age of participants with subjective voice problems was significantly lower than that of participants without such problems (*p*<0.0001). The mean sleep duration of all participants was 6.84±0.02 h/day. The mean sleep duration was significantly longer in those with than without dysphonia (*p =* 0.0056). The prevalence of dysphonia was higher in females (7.7%) than males (5.7%). All of age, gender, sleep duration, alcohol consumption, hypertension, metabolic syndrome, chronic kidney disease, occupation, and educational level were significantly associated with subjective voice problems.

**Table 1:** Analysis of factors potentially associated with dysphonia.

Parameter	Subjective Voice Problem (n = 17,806)		
	Yes (n = 1,218)	No (n = 16,588)	*P*-value
Age (years)	45.22±0.24	49.34±0.62	*<* .0001*
Gender_ men(%)	50.1(0.4)	39.2(1.8)	*<* .0001*
Body mass index (kg/m^2^)	23.69±0.04	23.8±0.14	.4365
Waist circumference (cm)	81.07±0.13	81.32±0.37	.4978
Sleep duration(hours/day)	6.85±0.02	6.69±0.06	*<* .0001*
Alcohol consumption(%)			
Non-drinker	24.6(0.5)	31.7(1.6)	
Mild to moderate drinker	65.4(0.5)	59.8(1.6)	
Heavy drinker	10.0(0.3)	8.4(1.1)	
Smoking(%)			.0894
Never smoker	56.7(0.5)	60.6(1.7)	
Ex-smoker	17.2 (0.4)	16.3(1.2)	
Current smoker	26.1(0.5)	23.1(1.6)	
Diabetes(%)	8.3(0.3)	9.1(0.9)	.4126
Hypertension(%)	27.0(0.5)	32.7(1.7)	.0005*
Metabolic syndrome(%)	25.7(0.5)	30.9(1.8)	.0026*
CKD(eGFR <60) (%)	6.1(0.2)	10.4(1.0)	*<* .0001*
Regular exercise (%)	19.6(0.5)	19.7(1.5)	.9256
Job_ working (%)	64.1(0.5)	61.1(1.7)	.0474*
Marital status_ with spouse (%)	80.0(0.7)	79.5(1.4)	.7038
Residential area_ urban (%)	80.0(1.7)	78.9(2.6)	.5479
Education; ≥ high (%)	71.4(0.7)	63.6(1.9)	*<* .0001*
Income; lowest quartile (%)	15.7(0.5)	23.3(1.6)	.5996

Values are presented as mean ± SE or %(SE).

* Significant at *p*<0.05

Abbreviation: CKD; chronic kidney disease, eGFR; estimated glomerular filtration rate

### Prevalence of subjective voice problems by sleep duration

**Fig 1:**
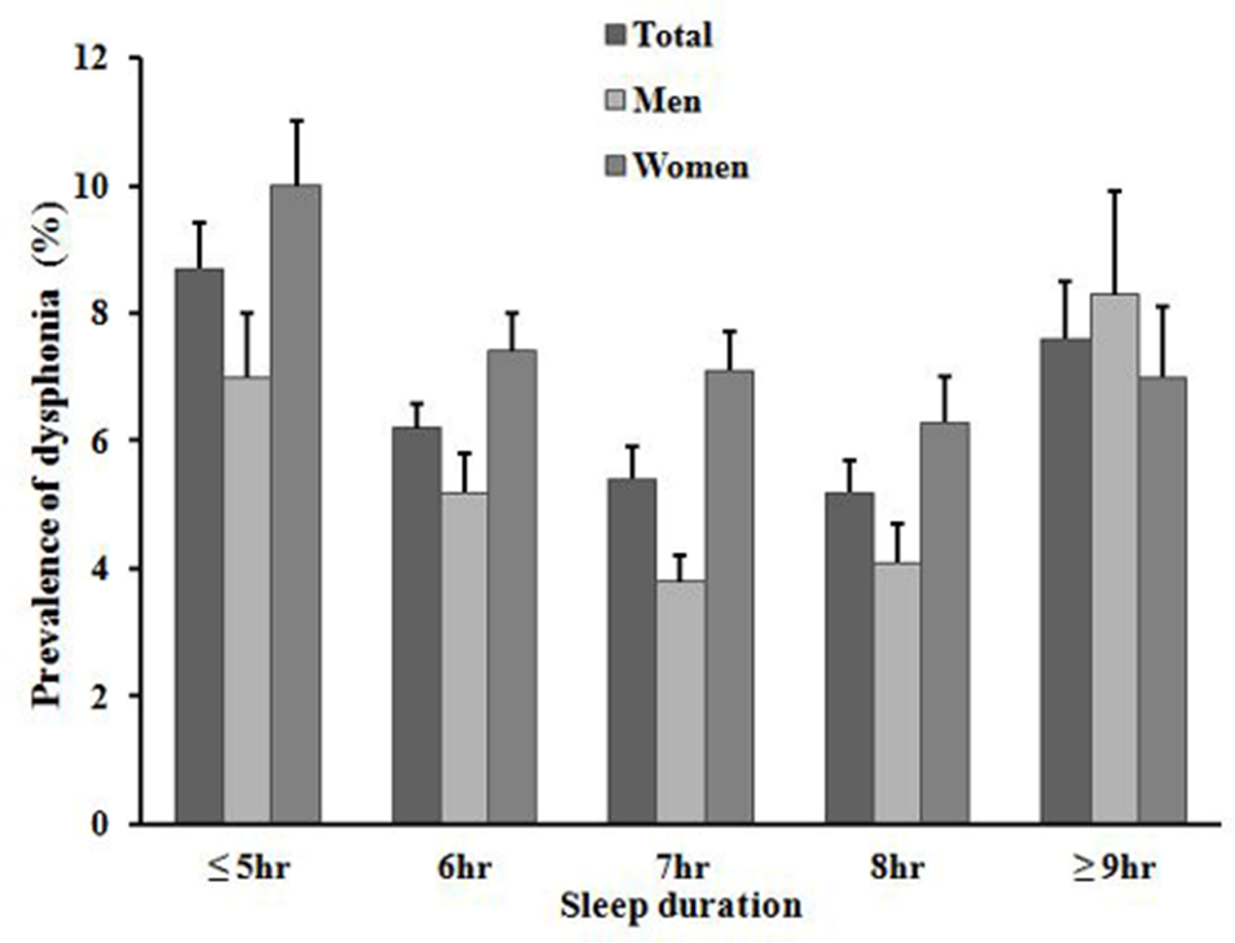
The prevalence of dysphonia by sleep duration.

Fig 1 shows the prevalence of subjective voice problems by sleep duration exhibited a U-shape, with the lowest point being at a sleep duration of 7-8h. The prevalence of dysphonia was significantly lower when the sleep duration was 7 h/day (*p*<0.0001, 0.0003, and 0.0055 for all subjects, males, and females, respectively). Male dysphonia was most prevalent in those who slept ≥9 h/day, and lowest at a sleep duration of 7 h/day. For females, the figures were ≤ 5 h/day and 8 h/day.

### Multivariable analysis of associations between subjective voice problems and sleep duration

Odds ratios and 95% CIs were obtained by multivariable logistic regression. The risks of dysphonia in the five subgroups categorized by sleep duration were calculated. Table 2 shows that dysphonia was significantly associated with sleep duration. After adjustment for covariates (age, sex, smoking status, alcohol consumption, regular exercise, low income, and high-level education), both a sleep duration of ≤5 h/day (OR = 1.454, 95% CI, 1.153–1.832) and a sleep duration of ≥9 h/day (OR = 1.365, 95% CI, 1.017–1.832) were significantly associated with the development of dysphonia, compared to the reference sleep duration (7 h). However, dysphonia was significantly more likely to be associated with sleep duration in males. In particular, males who slept for ≤5 h/day, ≥9 h/day were at a 1.69 fold (OR = 1.649, 95% CI, 1.12–2.53), 2-fold (OR = 2.028, 95% CI, 1.22–3.35) higher odds of dysphonia, respectively. Only females who slept for ≤5 h/day were at a 1.34-fold (OR = 1.340, 95% CI, 1.020–1.760) higher odds. These figures did not change when a sleep duration of 8 h was used as the reference duration.

**Table 2:** Adjusted odds ratios of dysphonia according to sleep duration.

	Odds ratio(95% confidence intervals)		
Sleep duration	Model 1	Model 2	Model 3
**Total (n = 17,806)**			
≤5 hrs	1.437(1.145–1.803)*	1.421(1.131–1.785)*	1.454(1.153–1.832)*
6 hrs	1.158(0.954–1.406)	1.156(0.952–1.405)	1.426(1.017–2.000)*
7 hrs	1	1	1
8 hrs	0.965(0.772–1.205)	0.962(0.770–1.202)	0.954(0.764–1.191)
≥9 hrs	1.398(1.042–1.876)*	1.406(1.047–1.888)*	1.365(1.017–1.832)*
**Men (n = 7,578)**			
≤5 hrs	1.651(1.113–2.448)*	1.649(1.113–2.445)*	1.686(1.124–2.529)*
6 hrs	1.426(1.017–2.000)*	1.425(1.016–2.001)*	1.444(1.032–2.021)*
7 hrs	1	1	1
8 hrs	1.053(0.735–1.510)	1.051(0.734–1.504)	1.027(0.717–1.471)
≥9 hrs	2.124(1.308–3.451)*	2.116(1.297–3.450)*	2.028(1.227–3.352)*
**Women (n = 10,228)**			
≤5 hrs	1.355(1.043–1.76)*	1.330(1.023–1.730)*	1.340(1.020–1.760)*
6 hrs	1.025(0.815–1.289)	1.021(0.812–1.284)	1.013(0.802–1.280)
7 hrs	1	1	1
8 hrs	0.889(0.672–1.176)	0.889(0.671–1.178)	0.889(0.668–1.183)
≥9 hrs	1.002(0.699–1.437)	1.011(0.705–1.451)	0.967(0.674–1.389)

Model 1 was adjusted for age, sex

Model 2 was adjusted for age, sex, and BMI

Model 3 was adjusted for age, sex, smoke, alcohol, exercise, income, and education.

* Significant at *P*< 0.05

### Association between long-term dysphonia (≥3weeks) and sleep duration

**Fig 2:**
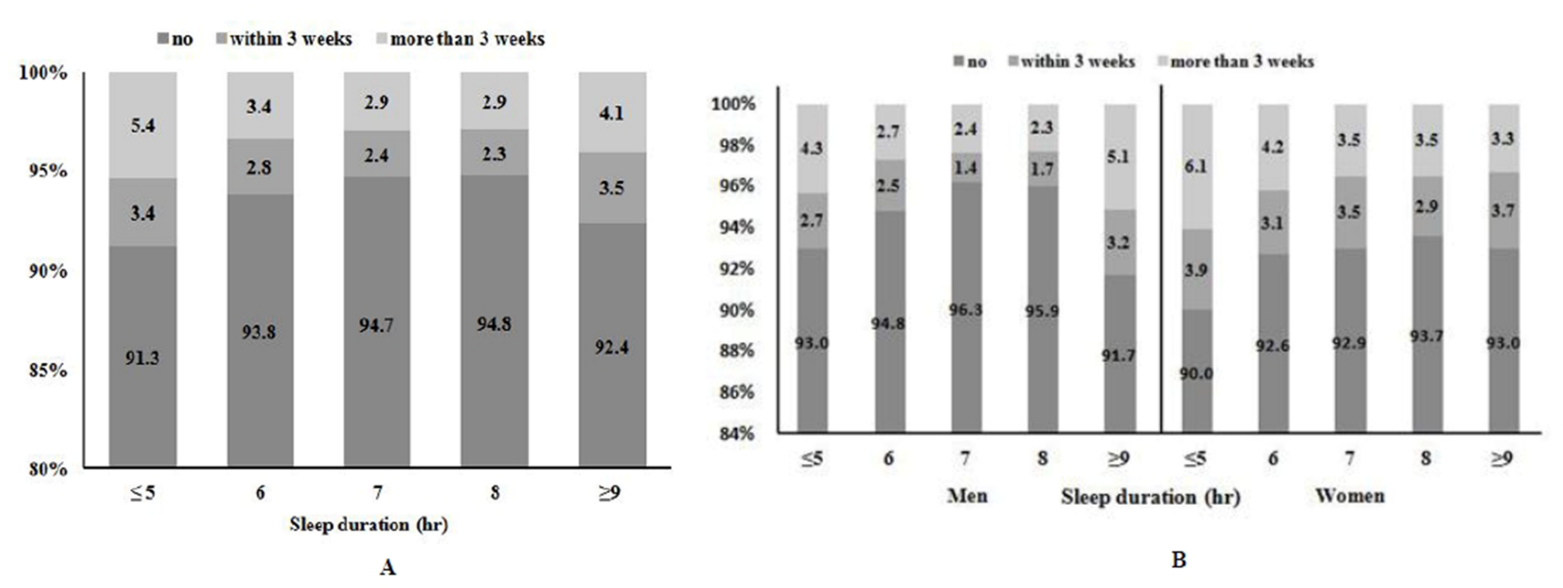
Distribution of dysphonia by sleep duration, stratified by duration of symptom. (A) Data for all participants and (B) data for men and women.

**Fig 3:**
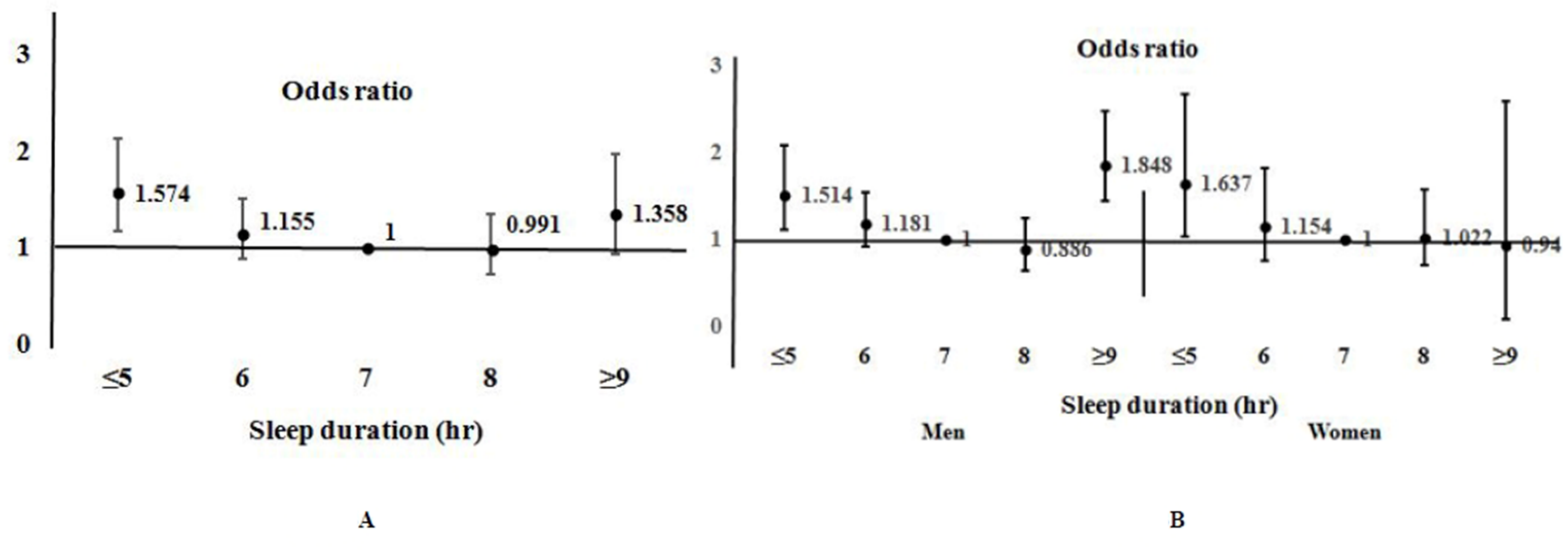
The odds ratio of dysphonia that lasts more than three weeks by sleep duration. (A) Data for all participants and (B) data for men and women.

The distribution of persistent dysphonia (≥3 weeks) by sleep duration is shown in Fig 2. The prevalence of short-term and long-term dysphonia differed significantly by sleep duration (*p*<0.0001). The overall prevalence of long-term dysphonia was 3.2%. The graph of [dysphonia ≥3 weeks in duration] against [sleep hours] was U-shaped, with a nadir at a sleep duration of 7–8 h. After analysing by gender, the U-shaped distribution of persistent dysphonia by sleep duration was shown in men but not women. When adjusted for covariates (age, sex, smoking status, alcohol consumption, regular exercise, low income, and high-level education), the odds ratio changed (Fig 3). The odds ratio for dysphonia ≥3 weeks in duration, graphed against sleep duration, was U-shaped, with the nadir at a sleep duration of 7–8 h. The odds ratio was 1.574 (95% CI, 1.203–2.247) for a sleep duration ≤5 h but 1.358 (95% CI, 0.912–2.642) for a sleep duration of ≥9 h. The odds of long-term dysphonia was thus higher when the sleep duration was short rather than long. The analysis by gender showed the U-shaped distribution of odds ratio for long-term dysphonia in men but not women.

## Discussion

This is the first population-based study to explore the associations between sleep duration and dysphonia in adults, by gender. Subjective voice problems were associated with abnormal sleep duration, especially in males. As self-perception of a vocal problem is always explored during a voice examination, most previous epidemiological studies on voice disorders have focused on subjective symptoms [18–22]. Dysphonia is any alteration in voice production, and is self-perceived. Dysphonia has many etiologies. Rosen et al. classified voice disorders into four major categories; organic, functional, movement, and systemic [23].

The possibility that sleep duration might influence the development of dysphonia or voice disorders has not previously been investigated. This is thus the first study to use multivariate analysis to show that sleep duration is significantly associated with dysphonia. Sleep duration may be abnormally short or long. Although the definitions of abnormal sleep duration might vary among papers, by different nations, cultures, and so on, short sleep has been usually defined as <7 h on average and long sleep as ≥9 h [2]. Both short and long sleepers are more likely to be in poorer overall health and to have been diagnosed with more medical conditions than normal sleepers [1–4]. Appropriate sleep is important in terms of both general health and quality of life. In our present population-based cross-sectional study, we found that the mean sleep duration was significantly longer in participants with than without dysphonia. Surprisingly, it also appears that long sleep duration is associated with dysphonia. After adjusting for confounders, longer sleep duration (≥ 9 h/day) showed the increased association with dysphonia. Although the reason for this association is unclear, males who slept longer were at a 2.0-fold higher odds of dysphonia than were those who slept for 7 h. Long sleep duration (≥9 h/day) has been associated with many sleep, medical, neurological, and psychiatric disorders [24]. According to previous reports, long sleep duration may mediate inflammatory, metabolic, or immune responses related to the risk of respiratory diseases [25, 26]. Therefore, we assumed that inflammatory or immune response of larynx can affect voice production and might induce dysphonia in long sleep duration.

Laryngeal disease is more common in males and subjective voice problems more common in females [27, 28]. Similarly, we found that dysphonia was more common in females than males. It was hypothesized that between-gender structural differences in laryngeal anatomy render females more vulnerable to voice disorders; the female vocal folds are shorter than those of males and have less hyaluronic acid(essential for wound repair) in the superficial layer of the lamina propria [28, 29].

Murry et al. showed that voice quality measured using the GRBAS scale (a perceptual rating of dysphonia severity) was strongly correlated with quality-of-life as measured using the V-RQOL(voice-related quality of life) instrument, especially in subjects aged <66 years [30]. Dysphonia severity was negatively associated with voice-related quality of life assessed using a voice symptom scale [31]. In particular, long-term dysphonia had a profound negative impact on the quality-of-life [13]. Although many voice problems are acute self-limiting infectious processes, voice problems persisting for >3 weeks are usually considered to be chronic and associated with various predisposing factors, including vocal fold mucosal disease, other medical conditions, and/or a neurogenic voice disorder [14]. The most common cause of community-acquired dysphonia is viral laryngitis, which persists for 1–3 weeks. Dysphonia persisting for longer requires further evaluation to ensure that no malignancy or other morbid condition has been missed, and to allow treatment of the specific vocal pathology, if indicated [14]. In the present study, we calculated adjusted odds ratios for long-term dysphonia by sleep duration. Compared to 7 h of sleep, a sleep duration of ≤5 h/day was associated with a 1.6-fold increased odds of development of long-term dysphonia. Thus, sleep deprivation may be associated with long-term dysphonia caused by significant vocal pathology.

One previous study found that fatigue is highly associated with functional dysphonia [32]. Fatigue may all affect behavior, leading to reduced activity, low mood, and reduced voice use [32]. Bagnall et al. found that fatigue caused by sleep deprivation triggered vocal changes, compromising the quality of vocal performance and contributing to the development of voice disorders [33]. In contrast to the (male-specific) association between excessive sleep and dysphonia, it was found that sleep deprivation was associated with dysphonia in both males and females. It is thus likely that sleep deprivation affects dysphonia development more than does excessive sleep; short sleep may be caused by insomnia, a psychiatric disorder, or obstructive sleep apnea (OSA). In particular, OSA may cause poor sleep and voice problems; bidirectional relationships have been found between OSA, on the one hand, and laryngeal sensory disturbances, laryngopharyngeal reflux, and a chronic cough, on the other [34–37]. OSA is a common sleep-related breathing disorder characterized principally by repetitive episodes of obstructive apnea and hypopnea during sleep [38]. Extraesophageal reflux (such as a laryngopharyngeal reflux) shares several risk factors with OSA; these are obesity, male sex, and alcohol use [38]. The symptoms of extraesophageal reflux vary and include regurgitation, heartburn, hoarseness, vocal fatigue, throat clearing, postnasal drip, cough, dysphagia, and globus. In addition, extraesophageal reflux is significantly more common in OSA patients than the general population. Eskiizmir et al. hypothesized that OSA and extraesophageal reflux may be related via a vicious cycle; an increased respiratory effort contributes to gastric acid reflux that, in turn, contributes to OSA progression by triggering inflammation that changes the upper airway mechanics via mucosal damage and sensory dysfunction [36]. Based on both previous data and our present results, it is clear that sleep disturbance, especially sleep deprivation, is associated with voice disorders.

A recent review article commented that functional dysphonia is associated with multiple psychosocial factors including anxiety, depression, and reduced quality of life [39]. Willinger et al. found that depression and anxiety symptoms were markedly more prevalent in patients with functional dysphonia [40]. According to recent meta-analysis, both short and long sleep duration is significantly associated with increased risk of depression in adults [41] Therefore, we presume that psychological factors, physical problems like fatigue and organic changes related to sleep problems or abnormal sleep duration may trigger vocal changes, compromise the quality of vocal performance, develop voice disorders and be finally associated with functional dysphonia.

In summary, considering above, it might be suggested that many diseases such as extraesophageal reflux, OSA and psychologic disorders, and inflammatory or immune response of larynx in relation with abnormal sleep time might affect voice production and yielded a U-shaped relationship between sleep duration and dysphonia.

Our study has certain limitations. First, we did not categorize the severity of self-reported voice problems as mild, moderate, or severe; we did not perform objective tests for analysing the quality of voice. Second, we did not use detailed or validated questionnaire on voice problems. Third, we did not seek to diagnose any laryngeal pathology; we did not identify subjects with infectious or reflux laryngitis (common causes of voice problems). Thus, we did not differentiate voice problems by severity or type and perform subgroup analysis according to underlying diseases. Fourth, our study subdivided subjects in 5 subgroups based on sleep duration, independent of the underlying cause of the sleep limitation, such decision means that we did not take into consideration specific co-morbidities such as gastro-esophageal reflux, OSA, parasomnia, presence of any organic disease, and so on, which might bridge the possible gap between sleep duration and voice disorder. Fifth, some related risk factors with dysphonia, such as voice overuse, occupation, medication, and environmental status, were not evaluated in this study because KNHANES did not include such information. Lastly, cross-sectional study cannot identify causal relationships. Thus, future longitudinal studies are required. However, the major strength of our work is that after adjusting for many confounders, we are the first to demonstrate an association between abnormal sleep duration and dysphonia.

## Conclusions

A U-shaped association is evident between sleep duration and mortality; we found a similar relationship between dysphonia and abnormal sleep duration in our population-based epidemiological study. Abnormally short and long sleep played a more significant role in dysphonia development in Korean adult males, but not females. In addition, sleep deprivation had a greater impact than did excessive sleep on the development of long-term dysphonia. Accurate epidemiological information contributes to healthcare planning, the development of preventative screening projects, and the provision of rehabilitative services. Our findings indicate that sleep disturbances should be controlled to prevent development of voice disorders. The mechanisms underlying the associations that we found should be studied further.
